# Supplementary material for: Genome-scale model of Rothia mucilaginosa predicts gene essentialities and reveals metabolic capabilities
Source: Microbiol Spectr. 2024 Apr 23;12(6):e04006-23. doi: 10.1128/spectrum.04006-23 (PMC11237427; doi:10.1128/spectrum.04006-23)
Supplement: Figure S1 — Experimentally-derived catabolic phenome of R. mucilaginosa DSM20746. [file spectrum.04006-23-s0001.pdf]

Figure S1: Experimentally-derived catabolic phenome of *R. mucilaginosa* DSM20746. The OD values were processed and analyzed as described in Materials and Methods. The heatmaps in Figure 5 facilitate the direct association of plate well labels with their corresponding compounds.

PM1

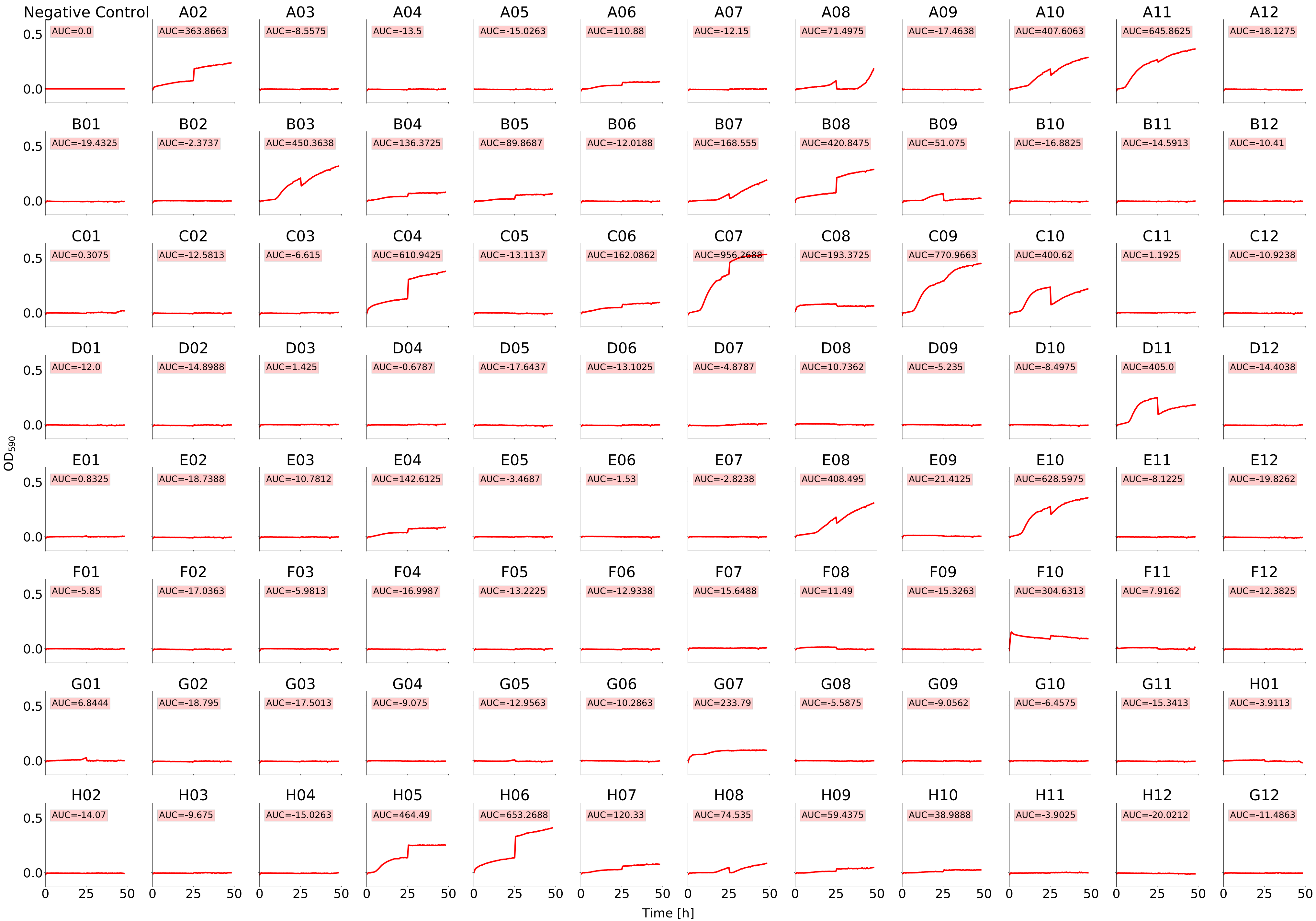
Negative Control
AUC=0.0
A02
AUC=363.8663
A03
AUC=-8.5575
A04
AUC=-13.5
A05
AUC=-15.0263
A06
AUC=110.88
A07
AUC=-12.15
A08
AUC=71.4975
A09
AUC=-17.4638
A10
AUC=407.6063
A11
AUC=645.8625
A12
AUC=-18.1275
B01
AUC=-19.4325
B02
AUC=-2.3737
B03
AUC=450.3638
B04
AUC=136.3725
B05
AUC=89.8687
B06
AUC=-12.0188
B07
AUC=168.555
B08
AUC=420.8475
B09
AUC=51.075
B10
AUC=-16.8825
B11
AUC=-14.5913
B12
AUC=-10.41
C01
AUC=0.3075
C02
AUC=-12.5813
C03
AUC=-6.615
C04
AUC=610.9425
C05
AUC=-13.1137
C06
AUC=162.0862
C07
AUC=956.2688
C08
AUC=193.3725
C09
AUC=770.9663
C10
AUC=400.62
C11
AUC=1.1925
C12
AUC=-10.9238
D01
AUC=-12.0
D02
AUC=-14.8988
D03
AUC=1.425
D04
AUC=-0.6787
D05
AUC=-17.6437
D06
AUC=-13.1025
D07
AUC=-4.8787
D08
AUC=10.7362
D09
AUC=-5.235
D10
AUC=-8.4975
D11
AUC=405.0
D12
AUC=-14.4038
E01
AUC=0.8325
E02
AUC=-18.7388
E03
AUC=-10.7812
E04
AUC=142.6125
E05
AUC=-3.4687
E06
AUC=-1.53
E07
AUC=-2.8238
E08
AUC=408.495
E09
AUC=21.4125
E10
AUC=628.5975
E11
AUC=-8.1225
E12
AUC=-19.8262
F01
AUC=-5.85
F02
AUC=-17.0363
F03
AUC=-5.9813
F04
AUC=-16.9987
F05
AUC=-13.2225
F06
AUC=-12.9338
F07
AUC=15.6488
F08
AUC=11.49
F09
AUC=-15.3263
F10
AUC=304.6313
F11
AUC=7.9162
F12
AUC=-12.3825
G01
AUC=6.8444
G02
AUC=-18.795
G03
AUC=-17.5013
G04
AUC=-9.075
G05
AUC=-12.9563
G06
AUC=-10.2863
G07
AUC=233.79
G08
AUC=-5.5875
G09
AUC=-9.0562
G10
AUC=-6.4575
G11
AUC=-15.3413
H01
AUC=-3.9113
H02
AUC=-14.07
H03
AUC=-9.675
H04
AUC=-15.0263
H05
AUC=464.49
H06
AUC=653.2688
H07
AUC=120.33
H08
AUC=74.535
H09
AUC=59.4375
H10
AUC=38.9888
H11
AUC=-3.9025
H12
AUC=-20.0212
G12
AUC=-11.4863
0.5
0.0
OD590
0
25
50
Time [h]

PM2A

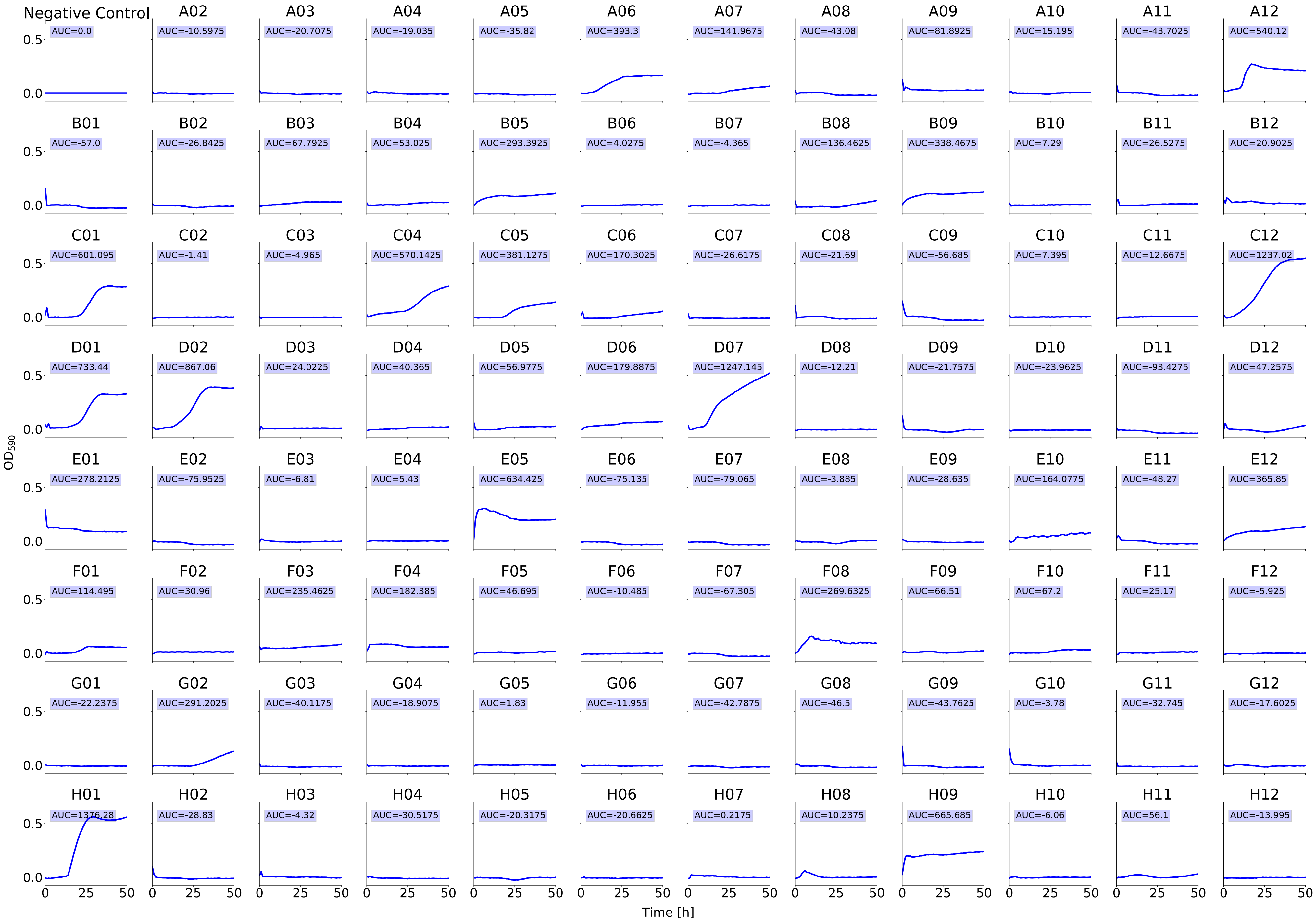

Negative Control
AUC=0.0
A02
AUC=-10.5975
A03
AUC=-20.7075
A04
AUC=-19.035
A05
AUC=-35.82
A06
AUC=393.3
A07
AUC=141.9675
A08
AUC=-43.08
A09
AUC=81.8925
A10
AUC=15.195
A11
AUC=-43.7025
A12
AUC=540.12
B01
AUC=-57.0
B02
AUC=-26.8425
B03
AUC=67.7925
B04
AUC=53.025
B05
AUC=293.3925
B06
AUC=4.0275
B07
AUC=-4.365
B08
AUC=136.4625
B09
AUC=338.4675
B10
AUC=7.29
B11
AUC=26.5275
B12
AUC=20.9025
C01
AUC=601.095
C02
AUC=-1.41
C03
AUC=-4.965
C04
AUC=570.1425
C05
AUC=381.1275
C06
AUC=170.3025
C07
AUC=-26.6175
C08
AUC=-21.69
C09
AUC=-56.685
C10
AUC=7.395
C11
AUC=12.6675
C12
AUC=1237.02
D01
AUC=733.44
D02
AUC=867.06
D03
AUC=24.0225
D04
AUC=40.365
D05
AUC=56.9775
D06
AUC=179.8875
D07
AUC=1247.145
D08
AUC=-12.21
D09
AUC=-21.7575
D10
AUC=-23.9625
D11
AUC=-93.4275
D12
AUC=47.2575
E01
AUC=278.2125
E02
AUC=-75.9525
E03
AUC=-6.81
E04
AUC=5.43
E05
AUC=634.425
E06
AUC=-75.135
E07
AUC=-79.065
E08
AUC=-3.885
E09
AUC=-28.635
E10
AUC=164.0775
E11
AUC=-48.27
E12
AUC=365.85
F01
AUC=114.495
F02
AUC=30.96
F03
AUC=235.4625
F04
AUC=182.385
F05
AUC=46.695
F06
AUC=-10.485
F07
AUC=-67.305
F08
AUC=269.6325
F09
AUC=66.51
F10
AUC=67.2
F11
AUC=25.17
F12
AUC=-5.925
G01
AUC=-22.2375
G02
AUC=291.2025
G03
AUC=-40.1175
G04
AUC=-18.9075
G05
AUC=1.83
G06
AUC=-11.955
G07
AUC=-42.7875
G08
AUC=-46.5
G09
AUC=-43.7625
G10
AUC=-3.78
G11
AUC=-32.745
G12
AUC=-17.6025
H01
AUC=1376.28
H02
AUC=-28.83
H03
AUC=-4.32
H04
AUC=-30.5175
H05
AUC=-20.3175
H06
AUC=-20.6625
H07
AUC=0.2175
H08
AUC=10.2375
H09
AUC=665.685
H10
AUC=-6.06
H11
AUC=56.1
H12
AUC=-13.995
OD590
Time [h]

PM3B

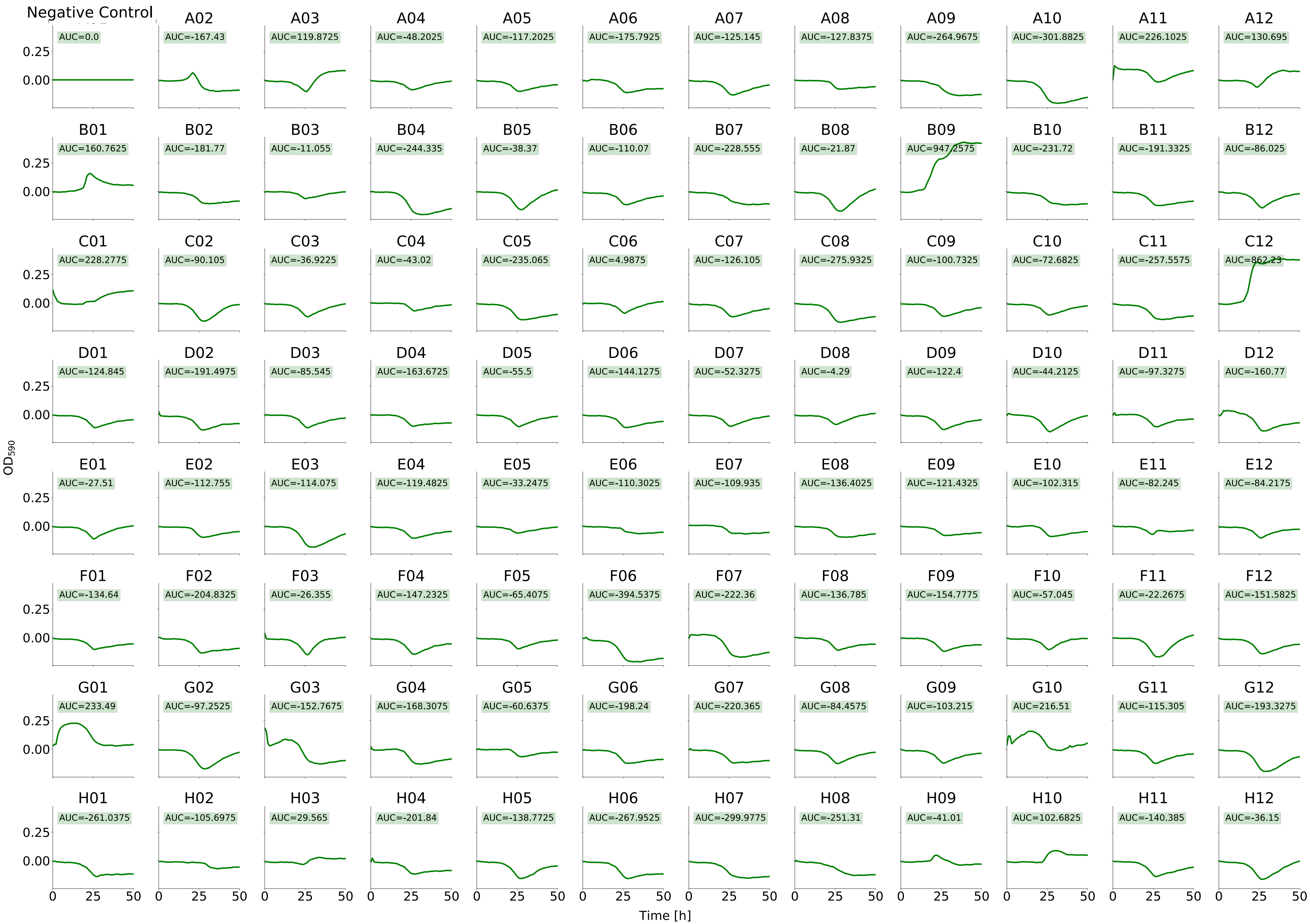
Negative Control
AUC=0.0
A02
AUC=-167.43
A03
AUC=119.8725
A04
AUC=-48.2025
A05
AUC=-117.2025
A06
AUC=-175.7925
A07
AUC=-125.145
A08
AUC=-127.8375
A09
AUC=-264.9675
A10
AUC=-301.8825
A11
AUC=226.1025
A12
AUC=130.695
B01
AUC=160.7625
B02
AUC=-181.77
B03
AUC=-11.055
B04
AUC=-244.335
B05
AUC=-38.37
B06
AUC=-110.07
B07
AUC=-228.555
B08
AUC=-21.87
B09
AUC=947.2575
B10
AUC=-231.72
B11
AUC=-191.3325
B12
AUC=-86.025
C01
AUC=228.2775
C02
AUC=-90.105
C03
AUC=-36.9225
C04
AUC=-43.02
C05
AUC=-235.065
C06
AUC=4.9875
C07
AUC=-126.105
C08
AUC=-275.9325
C09
AUC=-100.7325
C10
AUC=-72.6825
C11
AUC=-257.5575
C12
AUC=862.23
D01
AUC=-124.845
D02
AUC=-191.4975
D03
AUC=-85.545
D04
AUC=-163.6725
D05
AUC=-55.5
D06
AUC=-144.1275
D07
AUC=-52.3275
D08
AUC=-4.29
D09
AUC=-122.4
D10
AUC=-44.2125
D11
AUC=-97.3275
D12
AUC=-160.77
E01
AUC=-27.51
E02
AUC=-112.755
E03
AUC=-114.075
E04
AUC=-119.4825
E05
AUC=-33.2475
E06
AUC=-110.3025
E07
AUC=-109.935
E08
AUC=-136.4025
E09
AUC=-121.4325
E10
AUC=-102.315
E11
AUC=-82.245
E12
AUC=-84.2175
F01
AUC=-134.64
F02
AUC=-204.8325
F03
AUC=-26.355
F04
AUC=-147.2325
F05
AUC=-65.4075
F06
AUC=-394.5375
F07
AUC=-222.36
F08
AUC=-136.785
F09
AUC=-154.7775
F10
AUC=-57.045
F11
AUC=-22.2675
F12
AUC=-151.5825
G01
AUC=233.49
G02
AUC=-97.2525
G03
AUC=-152.7675
G04
AUC=-168.3075
G05
AUC=-60.6375
G06
AUC=-198.24
G07
AUC=-220.365
G08
AUC=-84.4575
G09
AUC=-103.215
G10
AUC=216.51
G11
AUC=-115.305
G12
AUC=-193.3275
H01
AUC=-261.0375
H02
AUC=-105.6975
H03
AUC=29.565
H04
AUC=-201.84
H05
AUC=-138.7725
H06
AUC=-267.9525
H07
AUC=-299.9775
H08
AUC=-251.31
H09
AUC=-41.01
H10
AUC=102.6825
H11
AUC=-140.385
H12
AUC=-36.15
0.25
0.00
0
25
50
OD590
Time [h]

PM4A

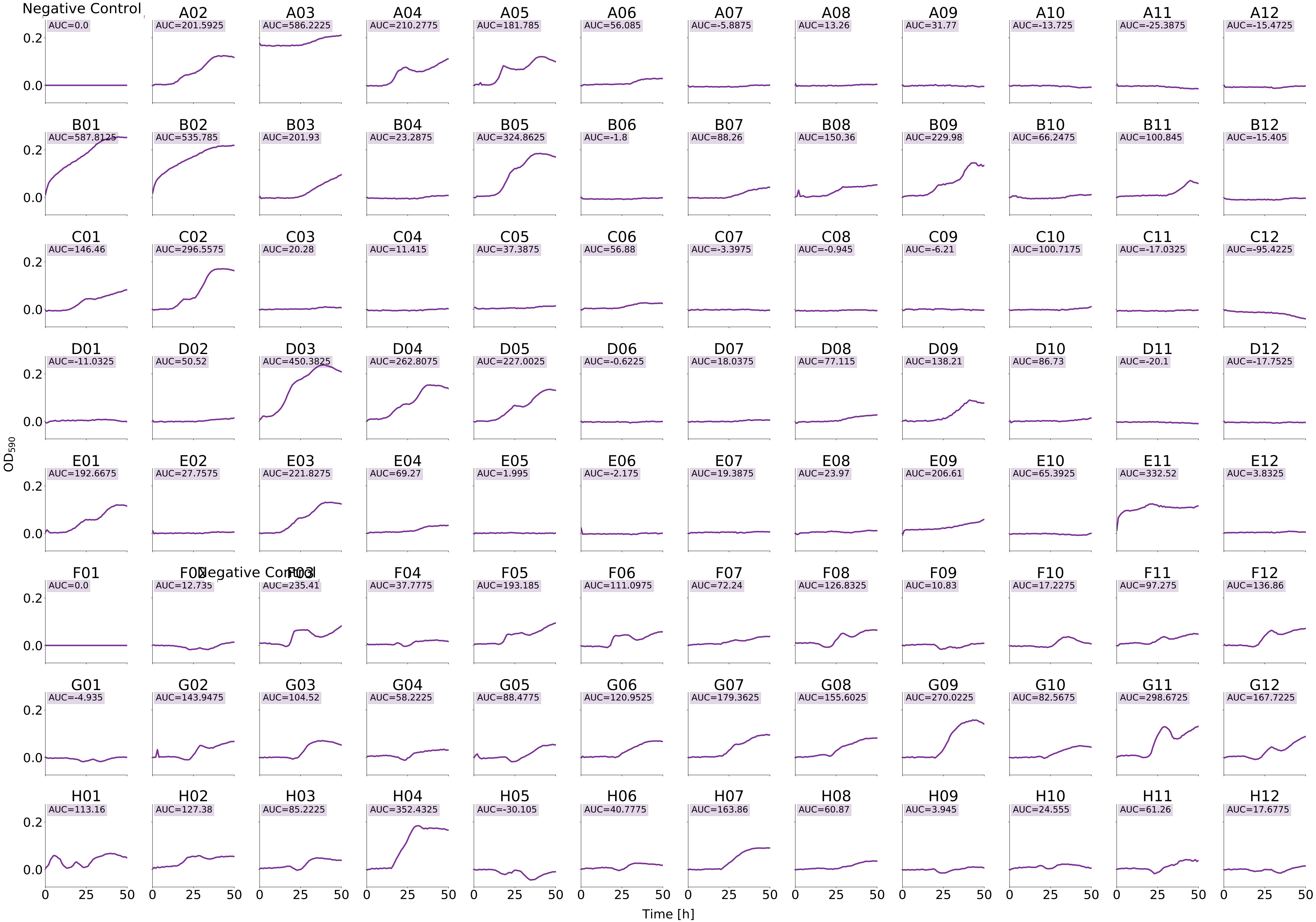
Negative Control
A02
A03
A04
A05
A06
A07
A08
A09
A10
A11
A12
AUC=0.0
AUC=201.5925
AUC=586.2225
AUC=210.2775
AUC=181.785
AUC=56.085
AUC=-5.8875
AUC=13.26
AUC=31.77
AUC=-13.725
AUC=-25.3875
AUC=-15.4725
B01
B02
B03
B04
B05
B06
B07
B08
B09
B10
B11
B12
AUC=587.8125
AUC=535.785
AUC=201.93
AUC=23.2875
AUC=324.8625
AUC=-1.8
AUC=88.26
AUC=150.36
AUC=229.98
AUC=66.2475
AUC=100.845
AUC=-15.405
C01
C02
C03
C04
C05
C06
C07
C08
C09
C10
C11
C12
AUC=146.46
AUC=296.5575
AUC=20.28
AUC=11.415
AUC=37.3875
AUC=56.88
AUC=-3.3975
AUC=-0.945
AUC=-6.21
AUC=100.7175
AUC=-17.0325
AUC=-95.4225
D01
D02
D03
D04
D05
D06
D07
D08
D09
D10
D11
D12
AUC=-11.0325
AUC=50.52
AUC=450.3825
AUC=262.8075
AUC=227.0025
AUC=-0.6225
AUC=18.0375
AUC=77.115
AUC=138.21
AUC=86.73
AUC=-20.1
AUC=-17.7525
E01
E02
E03
E04
E05
E06
E07
E08
E09
E10
E11
E12
AUC=192.6675
AUC=27.7575
AUC=221.8275
AUC=69.27
AUC=1.995
AUC=-2.175
AUC=19.3875
AUC=23.97
AUC=206.61
AUC=65.3925
AUC=332.52
AUC=3.8325
F01
F02
Negative Control
F03
F04
F05
F06
F07
F08
F09
F10
F11
F12
AUC=0.0
AUC=12.735
AUC=235.41
AUC=37.7775
AUC=193.185
AUC=111.0975
AUC=72.24
AUC=126.8325
AUC=10.83
AUC=17.2275
AUC=97.275
AUC=136.86
G01
G02
G03
G04
G05
G06
G07
G08
G09
G10
G11
G12
AUC=-4.935
AUC=143.9475
AUC=104.52
AUC=58.2225
AUC=88.4775
AUC=120.9525
AUC=179.3625
AUC=155.6025
AUC=270.0225
AUC=82.5675
AUC=298.6725
AUC=167.7225
H01
H02
H03
H04
H05
H06
H07
H08
H09
H10
H11
H12
AUC=113.16
AUC=127.38
AUC=85.2225
AUC=352.4325
AUC=-30.105
AUC=40.7775
AUC=163.86
AUC=60.87
AUC=3.945
AUC=24.555
AUC=61.26
AUC=17.6775
0.2
0.0
0
25
50
OD590
Time [h]
