# Supplementary material for: Genome-scale model of Rothia mucilaginosa predicts gene essentialities and reveals metabolic capabilities
Source: Microbiol Spectr. 2024 Apr 23;12(6):e04006-23. doi: 10.1128/spectrum.04006-23 (PMC11237427; doi:10.1128/spectrum.04006-23)
Supplement: Figure S2 — Growth curves of the independent confirmatory tests for validating the Biolog PM results. [file spectrum.04006-23-s0002.pdf]

Figure S2: Growth curves of the independent confirmatory tests for validating the Biolog PM results. The abbreviations used in the figure legend are as follows: Man: mannose, Met: L-methionine, Ado: adonitol, Orn: L-ornithine, Sal: salicin, Glc: α-D-glucose, Succ: succinate, Ala: l-alanine, His: L-histidine, and IF: inoculation fluid. The M9 pure medium was prepared according to the specifications outlined in Table S1, and individual substrates were supplemented at the concentrations detailed in Table S2. The corresponding Biolog results are presented in tabular format, classified by growth (G) and no growth (NG).

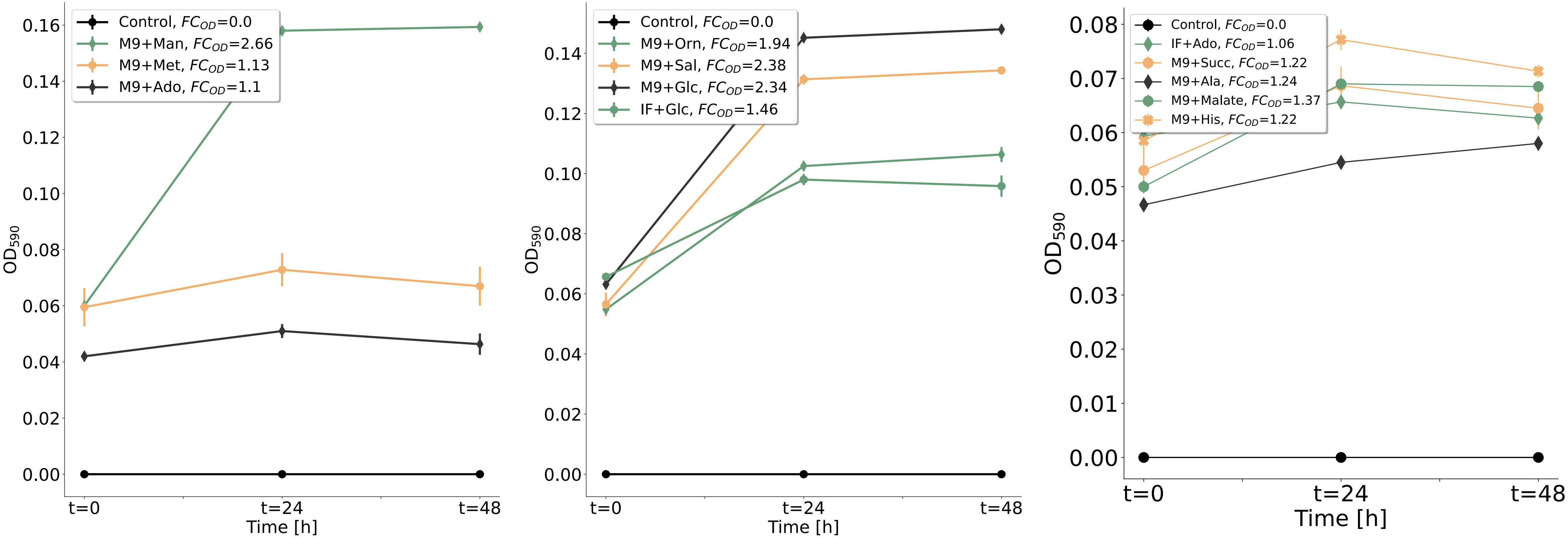

	**M9+Man**	**M9+Met**	**M9+Ado**	**M9+Orn**	**M9+Sal**	**M9+Glc**	**IF+Glc**
BIOLOG	G	NG	NG	G	G	G	G
Stat. test	***	ns	ns	*	**	***	*

	**IF+Ado**	**M9+Succ**	**M9+Ala**	**M9+Malate**	**M9+His**
BIOLOG	NG	NG	NG	NG	NG
Stat. test	ns	ns	ns	ns	ns
