# Supplementary material for: Genome-scale model of Rothia mucilaginosa predicts gene essentialities and reveals metabolic capabilities
Source: Microbiol Spectr. 2024 Apr 23;12(6):e04006-23. doi: 10.1128/spectrum.04006-23 (PMC11237427; doi:10.1128/spectrum.04006-23)
Supplement: Figure S3 — Detailed comparative analysis of gene essentiality in silico predictions using iRM23NL. [file spectrum.04006-23-s0003.pdf]

Figure S3: Detailed comparative analysis of gene essentiality in silico predictions using *i*RM23NL. Comparison of predicted essential genes using four nutrient environments (LB, M9 supplemented with glucose, SCFM, and SNM) under both oxic and anoxic conditions.

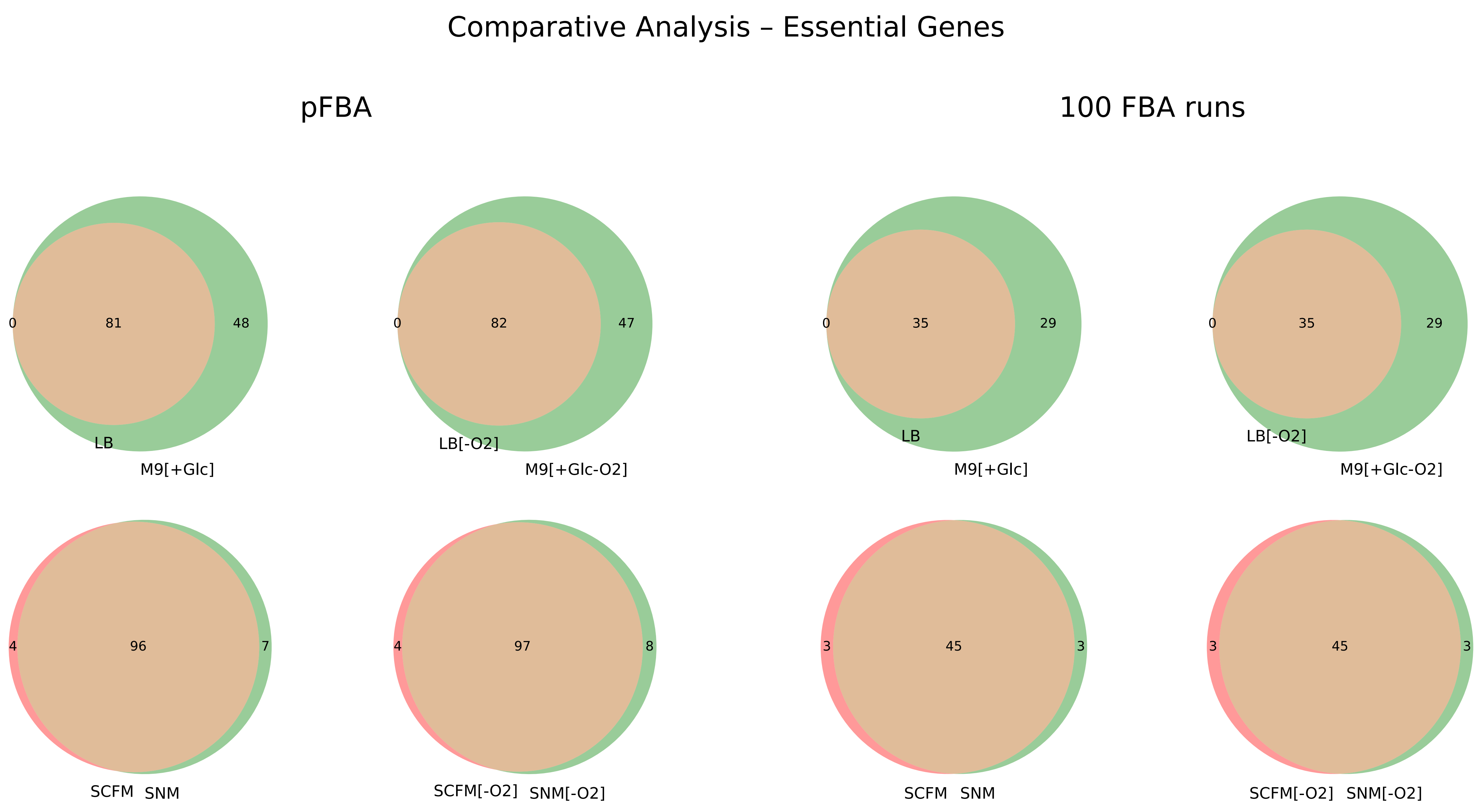
Comparative Analysis – Essential Genes
pFBA
100 FBA runs
0
81
48
LB
M9[+Glc]
0
82
47
LB[-O2]
M9[+Glc-O2]
0
35
29
LB
M9[+Glc]
0
35
29
LB[-O2]
M9[+Glc-O2]
4
96
7
SCFM
SNM
4
97
8
SCFM[-O2]
SNM[-O2]
3
45
3
SCFM
SNM
3
45
3
SCFM[-O2]
SNM[-O2]

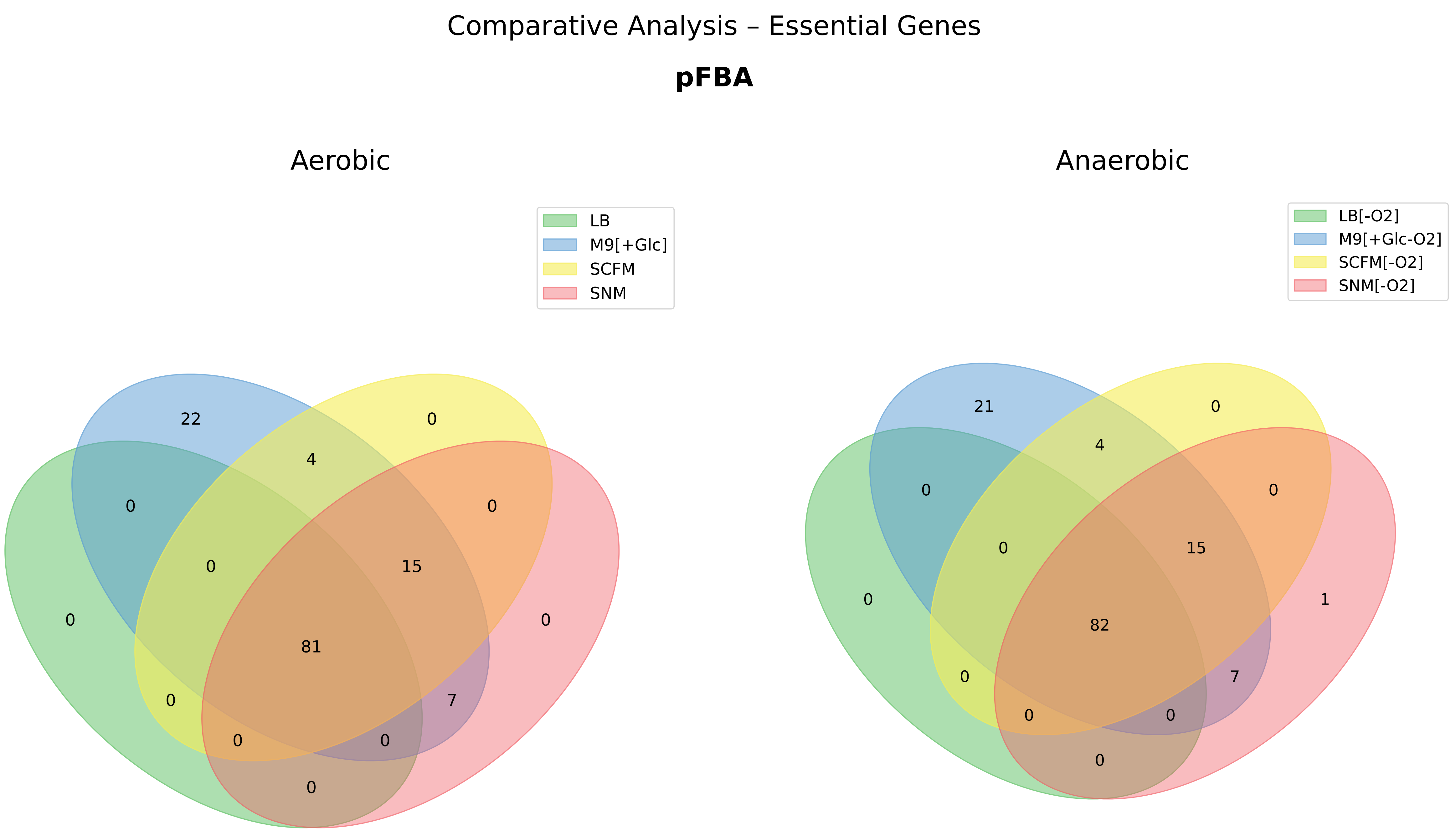
Comparative Analysis – Essential Genes
pFBA
Aerobic
LB
M9[+Glc]
SCFM
SNM
22
0
4
0
0
0
15
0
0
81
0
7
0
0
0
0
Anaerobic
LB[-O2]
M9[+Glc-O2]
SCFM[-O2]
SNM[-O2]
21
0
4
0
0
0
15
0
1
82
0
7
0
0
0
0

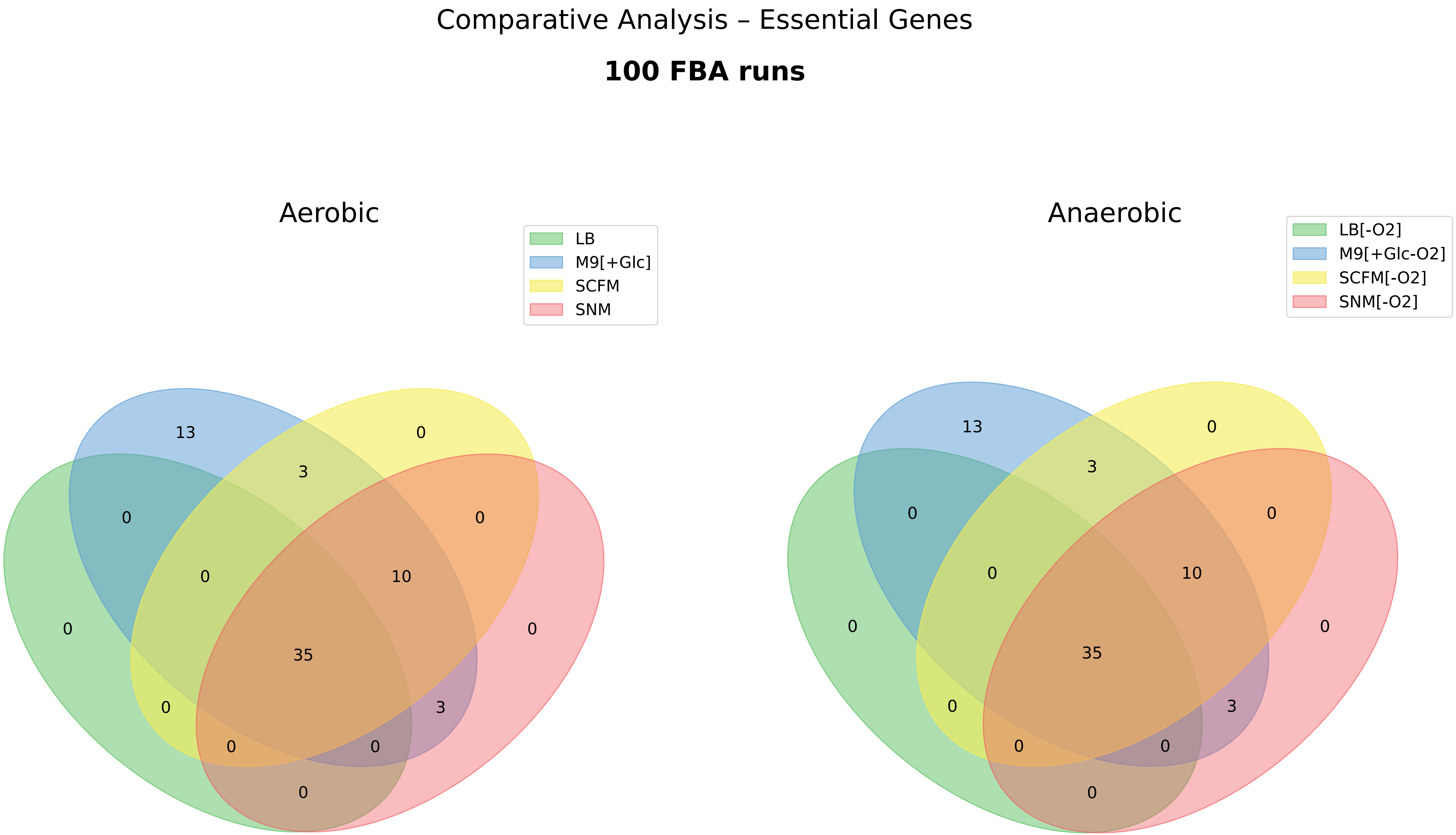
Comparative Analysis – Essential Genes
100 FBA runs
Aerobic
LB
M9[+Glc]
SCFM
SNM
13
0
3
0
0
10
0
0
35
0
3
0
0
0
0
Anaerobic
LB[-O2]
M9[+Glc-O2]
SCFM[-O2]
SNM[-O2]
13
0
3
0
0
10
0
0
35
0
3
0
0
0
0
