# Supplementary material for: Genome-scale model of Rothia mucilaginosa predicts gene essentialities and reveals metabolic capabilities
Source: Microbiol Spectr. 2024 Apr 23;12(6):e04006-23. doi: 10.1128/spectrum.04006-23 (PMC11237427; doi:10.1128/spectrum.04006-23)
Supplement: Figure S4 — Distribution of essential genes in metabolic subsystems. [file spectrum.04006-23-s0004.pdf]

S4 Figure. Distribution of essential genes in metabolic subsystems. The classification of *in silico*-predicted essential genes based on annotated Gene Ontology (GO) terms.

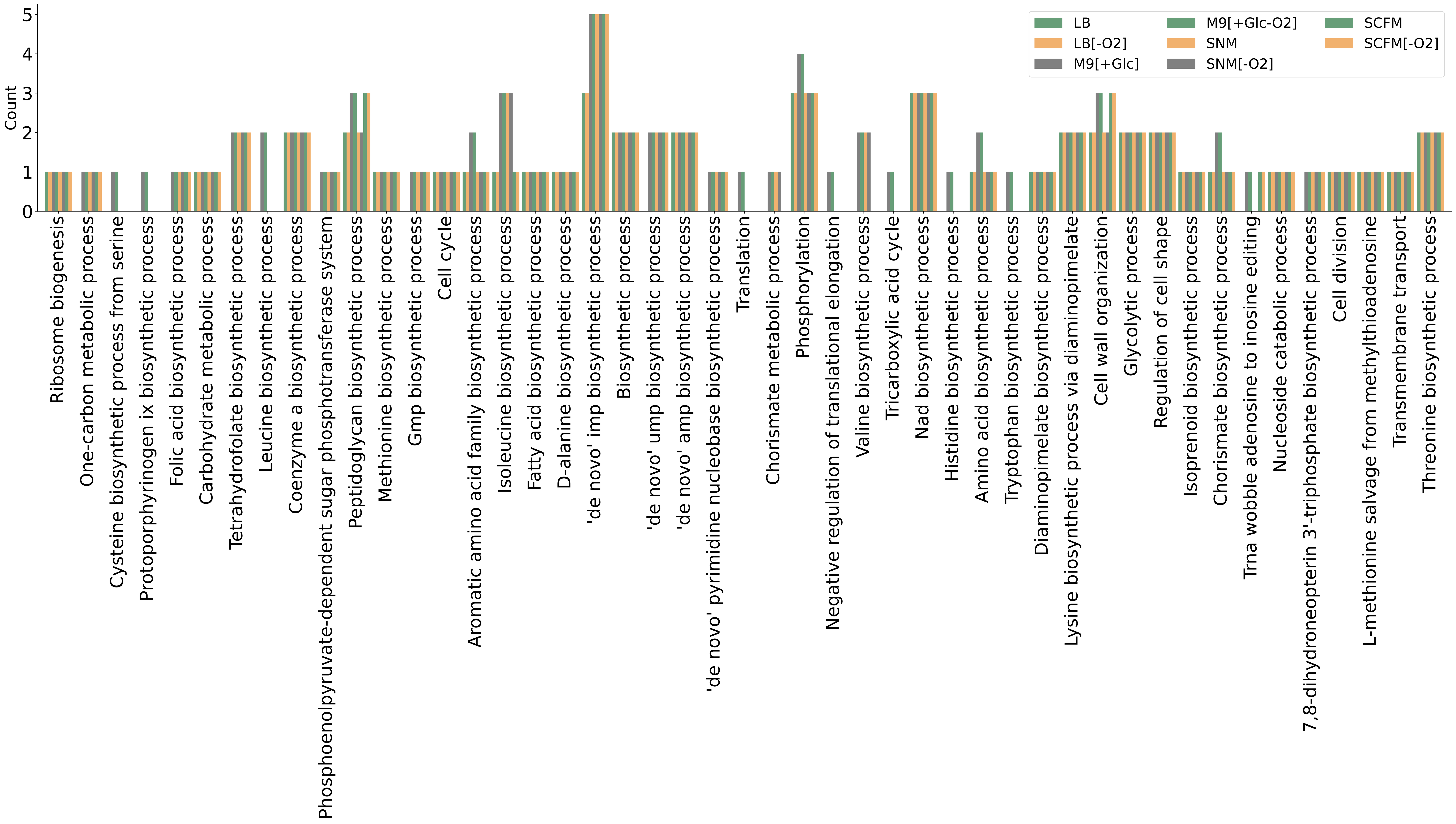
LB
LB[-O2]
M9[+Glc]
M9[+Glc-O2]
SNM
SNM[-O2]
SCFM
SCFM[-O2]
Count
Ribosome biogenesis
One-carbon metabolic process
Cysteine biosynthetic process from serine
Protoporphyrinogen ix biosynthetic process
Folic acid biosynthetic process
Carbohydrate metabolic process
Tetrahydrofolate biosynthetic process
Leucine biosynthetic process
Coenzyme a biosynthetic process
Phosphoenolpyruvate-dependent sugar phosphotransferase system
Peptidoglycan biosynthetic process
Methionine biosynthetic process
Gmp biosynthetic process
Cell cycle
Aromatic amino acid family biosynthetic process
Isoleucine biosynthetic process
Fatty acid biosynthetic process
D-alanine biosynthetic process
'de novo' imp biosynthetic process
Biosynthetic process
'de novo' ump biosynthetic process
'de novo' amp biosynthetic process
'de novo' pyrimidine nucleobase biosynthetic process
Translation
Chorismate metabolic process
Phosphorylation
Negative regulation of translational elongation
Valine biosynthetic process
Tricarboxylic acid cycle
Nad biosynthetic process
Histidine biosynthetic process
Amino acid biosynthetic process
Tryptophan biosynthetic process
Diaminopimelate biosynthetic process
Lysine biosynthetic process via diaminopimelate
Cell wall organization
Glycolytic process
Regulation of cell shape
Isoprenoid biosynthetic process
Chorismate biosynthetic process
Trna wobble adenosine to inosine editing
Nucleoside catabolic process
7,8-dihydroneopterin 3'-triphosphate biosynthetic process
Cell division
L-methionine salvage from methylthioadenosine
Transmembrane transport
Threonine biosynthetic process
